# Different skeletal protein toolkits achieve similar structure and performance in the tropical coral *Stylophora pistillata* and the temperate *Oculina patagonica*

**DOI:** 10.1038/s41598-022-20744-0

**Published:** 2022-10-04

**Authors:** Tal Zaquin, Anna Paola Di Bisceglie, Iddo Pinkas, Giuseppe Falini, Tali Mass

**Affiliations:** 1 Department of Marine Biology, Leon H. Charney School of Marine Sciences, University of Haifa, Mount Carmel, 3498838 Haifa, Israel; 2 Department of Chemistry “Giacomo Ciamician”, University of Bologna, Via F. Selmi 2, 40126 Bologna, Italy; 3 Department of Chemical Research Support, Weizmann Institute of Science, 76100 Rehovot, Israel

**Keywords:** Molecular evolution, Computational biology and bioinformatics, Marine biology, Protein sequencing, Mass spectrometry

## Abstract

Stony corals (order: Scleractinia) differ in growth form and structure. While stony corals have gained the ability to form their aragonite skeleton once in their evolution, the suite of proteins involved in skeletogenesis is different for different coral species. This led to the conclusion that the organic portion of their skeleton can undergo rapid evolutionary changes by independently evolving new biomineralization-related proteins. Here, we used liquid chromatography-tandem mass spectrometry to sequence skeletogenic proteins extracted from the encrusting temperate coral *Oculina patagonica*. We compare it to the previously published skeletal proteome of the branching subtropical corals *Stylophora pistillata* as both are regarded as highly resilient to environmental changes. We further characterized the skeletal organic matrix (OM) composition of both taxa and tested their effects on the mineral formation using a series of overgrowth experiments on calcite seeds. We found that each species utilizes a different set of proteins containing different amino acid compositions and achieve a different morphology modification capacity on calcite overgrowth. Our results further support the hypothesis that the different coral taxa utilize a species-specific protein set comprised of independent gene co-option to construct their own unique organic matrix framework. While the protein set differs between species, the specific predicted roles of the whole set appear to underline similar functional roles. They include assisting in forming the extracellular matrix, nucleation of the mineral and cell signaling. Nevertheless, the different composition might be the reason for the varying organization of the mineral growth in the presence of a particular skeletal OM, ultimately forming their distinct morphologies.

## Introduction

Stony corals produce huge masses of calcium carbonate structures in the ocean in the form of aragonite and are among the oldest biomineralizing Metazoa^1,2^. In addition, they exhibit high variance in growth forms within and between taxa, ranging from simple encrusting to tree-like branching forms^3^ and mediating the formation of massive reefs in tropical, sub-tropical and cold-water seas. These structures then provide a complex three-dimensional ecosystem that offers shelter and sanctuary to a range of marine life^4,5^. These complex ecosystems are facilitated by depositing an aragonitic skeleton with species-specific macromorphology that can vary along environmental gradients and locations^6^.

At the macro scale, the skeletons of scleractinian corals are morphologically diverse^3^; for example, massive as in *Favia*, branching as in *Stylophora*, encrusting as in *Oculina*, or table-like similarly to *Turbinaria*. However, this diversity is not evident at the micro-scale, where stony coral skeletons reveal textural similarities, displaying needle-like single aragonite crystal fibers radiating from centers of calcification (CoCs)^7–9^ and forming superstructures that are reported to have a spherulitic organization^10^.

Besides the aragonitic mineral, coral skeletons are composed of up to 2.5 w% water associated with organics molecules^10^. Those biomolecules, dubbed the skeletal organic matrix (OM), are secreted through the calicoblastic cell layer (the cell layer that deposits the coral skeleton). The skeletal OM is composed of polysaccharides, lipids, and proteins, where proteins are the most studied fraction, having critical roles in skeleton formation^2,11,12^ It has been suggested that corals' ability to control skeleton formation is exercised through the skeletal OM, whose biosynthesis is orchestrated in space and time by the activity of the calicoblastic cells^11^.

However, the exact function of only a few skeletal OM proteins is known to date^13^. Efforts to sequence stony coral skeletal OM proteins have revealed many seemingly unique proteins at both the family and species levels^14–19^. While many of these biomolecules differ between taxa, explorations of the evolutionary history of coral skeletal OM proteins from divergent coral genera have found that a minor portion of proteins is conserved across species, referred to as the "core molecular biomineralization toolkit^19^". Nevertheless, approximately half of the skeletal OM proteins were independently co-opted from ancestors shared with other phyla, some containing no extant skeleton-forming taxa^19^. While it appears that the specific elements to form a skeleton are diverse between species, there might be a conserved genetic basis for some of the shared microstructural aspects of coral skeleton formation^13^, as it was found in vivo and in vitro that the skeletal OM will bias the mineral polymorph toward aragonite^20,21^.

These features may influence the ongoing viability of the different species to withstand the combined challenge of global warming and ocean acidification. Therefore, it is of primary importance to study the formation of the skeleton in the extent of coral species, which exhibit different population organization and resilience to environmental stresses, like ocean acidification and increased temperature.

In this context, we investigated the sequences of the skeletal OM of an encrusting temperate coral, *Oculina patagonica,* which is endemic to the Mediterranean Sea^22^. We first used liquid chromatography-tandem mass spectrometry to characterize the protein composition of skeletal proteins extracted from the temperate coral *O. patagonica.* We then compared our results with previously published skeletal OM data of the branching sub-tropic corals: *Stylophora pistillata*^14,17^, *Acropora digitifera*^16^ and *A. millepora*^15^. The corals *O. patagonica* and *S. pistillata* belong to the Robusta coral clade, while both *A. digitifera* and *A. millepora* belong to the Complexa clade^23^. In addition, we compared the sequence composition of the two Robusta coral representatives, *O. patagonica* and *S. pistillata* and tested the effect of their soluble skeletal OM fraction (SOM) on the precipitation of CaCO_3_ on calcite seeds in vitro*.* We found that each Robusta species utilizes a different set of proteins, contains different amino acid compositions, and has a different morphology modification capacity on calcite overgrowth. Our results further support the hypothesis that the different coral species utilize a species-specific protein set of independent gene co-option to construct their own unique organic matrix framework.

## Materials and methods

### Materials

All chemicals were obtained from Merck©, were of analytical grade and were used without further purification. All glassware was cleaned in ethanol and rinsed with distilled water before being air-dried.

### Coral samples collection and preparation for protein extraction

Colonies of *O. patagonica* were collected in the Israeli Mediterranean Sea at Sdot-Yam (32° 49 N 34° 88 E) from 1 to 3 m depth. In addition, *S. pistillata* colonies were collected in the Israeli Red Sea, in front of the H. Steinitz Marine Biology Laboratory, Eilat (29° 30 N, 34° 56 E), from 3 to 5 m depth. Samples were collected under permit number 42410/2019 from the Israeli Natural Parks Authority.

Removal of organics tissue from the skeleton was done following the modified methods of Stoll et al.^17,24^. First, coral colonies were fragmented and oxidized with 20 ml 1:1 of 30% H_2_O_2_ and 3% NaClO solution while adding 1.5 ml of 3% NaClO every 20 min. After overnight incubation at room temperature, the solution was removed by washing the fragments five times for one minute with ultra-pure water and drying overnight at 60 °C. To ensure that no organic residue remained, we crushed the fragments to ≤ 63 µm in diameter with a mortar and pestle, oxidized, and washed them in ultra-pure water three more times. Between each cycle, both solutions were removed from the skeletal powder by centrifugation at 5000×*g* for 3 min at 4 °C and dried overnight at 60 °C.

### Extraction and purification of skeletal proteins

Approximately 1.5 g of cleaned skeleton powder was used from each sample to extract the coral skeletal proteins, using the "CF4" method described in Peled et al.^17^. In brief, samples were decalcified in 0.5 M acetic acid for three hours at room temperature in Falcon tubes. Next, the samples were centrifuged at 5000×*g* for 5 min at 4 °C, and the supernatant was stored at 4 °C. The undissolved pellets were further treated until decalcification was completed, around three rounds, and until the measured pH in the solution was ~ 6. Next, the samples were frozen overnight at − 80 °C, lyophilized until dry and later merged by resuspension in 10 ml of ultra-pure water. The merged samples were frozen overnight at − 80 °C and lyophilized until dry. Next, the lyophilized pellet was resuspended in 12 ml ultra-pure water and centrifuged on a 3 kDa cutoff Amicon® Ultra 15 centrifugal filter units (Merck©) at 5000×*g* (4 °C) to a 0.5 ml final volume of desalted and concentrated skeletal OM. This process was repeated twice (three rounds in total), and the solution was separated into soluble and insoluble fractions by centrifugation at 5000×*g* for 5 min at 4 °C. The skeletal OM from each species was characterized by amino acid analysis and Fourier-transform infrared spectroscopy (FTIR) (see SI). The skeletal OM concentration (µg/ml) was expressed as the amount of protein from the amino acid analysis^25^.

### *O. patagonica* proteomic analysis

#### Protein sequencing

*O. patagonica* skeletal protein samples were sequenced using the S-trap method^26^, where the resulting peptides were analyzed using a nanoflow liquid chromatography (nanoAcquity) coupled with a mass spectrometer (Fusion Lumos) (see SI). The Byonic search engine (Protein Metrics Inc.) was used to examine the resulting data against the predicted proteins from a de novo transcriptome of *O. patagonica*^27^ and a common contaminants database. First, no false discovery rate (FDR) filtering was implemented in the examination to generate a focused database for a second search. Next, the FDR was set to 1%, allowing fixed carbamidomethylation on C and variable oxidation on molecular weight, deamidation on NQ and protein N-terminal acetylation.

#### Data sorting

We used the predicted proteins *O. patagonica*^27^ as a reference peptide database for the MS analysis. We also included a common contaminants database. Only proteins with at least two significant peptides or at least one significant peptide with at least ten spectra and an identification score of 250 or greater were retained. To further filter out potential human proteins inadvertently introduced during sample preparation, we used the filtering criteria in Peled et al.^17^. In brief, all sequences were BLASTed against the 'Primates' nr database in NCBI using the Blast + command line (2.10.1)^28^. Lastly, we identified and removed from our analysis the BLAST sequence alignments of scleractinian versus Homo sapiens proteins with e-values lower than e^−50^ and percent mean similarity greater than 50% and sequences with e-values lower than e^−100^.

### Characterization and annotation of SOM protein sequences

*O. patagonica*'s protein sequences identified through the proteomic analysis were annotated using the Trinotate pipeline^29^.

The orthologous relationship between the species was determined using OrthoFinder 2.5.2^30,31^. As sufficient species sampling is required to infer orthologous relationships between species, we sampled 12 species from the scleractinian order with an annotated genome supplemented with a combination of diverse metazoans from public databases (Table SI3). OrthoFinder generates orthology groups (Orthogroups) based on normalized reciprocal best BLAST hits' bit scores and then estimates orthologues genes pairs within Orthogroups. We then selected all pairs of *O. patagonica* skeletal OM sequences orthologous to *S. pistillata* SOM sequences (1:1, 1:many, many:many relationships). It is noteworthy that the use of de novo transcriptomes in inferring orthology is not recommended. However, the combination of a transcriptomic database and the species proteome is robust for accurately identifying proteins.

Nevertheless, another concern might be identifying multiple isoforms and transcripts of the same fragmented gene. To overcome this ambiguity, we have manually reviewed each rooted gene tree produced by OrthoFinder and their respective multiple sequence alignments (MSA) where skeletal OM proteins were identified. We examined all terminal nodes resulting from predicted duplication events to identify multiple isoforms classified as different skeletal OM proteins. In cases where the sequences aligned at over a 90% similarity, we considered them redundant and only kept the isoform with better MS evidence. In cases where the transcripts did not align with each other, the closest sequence (derived from a speciation event) was used as a scaffold to align the transcript as they were considered to be fragments of the same protein. A presence-absence (PA) matrix was generated based on orthologous sequences identified between at least two species out of *O. patagonica, S. pistillata, A. digitifera* And *A. millepora* (Table SI5). Non-metric multidimensional scaling (NMDS) was calculated based on the Jaccard distance matrix using the PA matrix.

Skeletal OM protein sequences from both *O. patagonica* (this study) and *S. pistillata*^14,17^ were analyzed using the InterProScan 5.50 platform^32^ in order to find conserved functional domains (Pfam)^33^ and gene ontology (GO) terms^34^. Using the InterPro predictive information, each skeletal OM protein domain and sequence were categorized into functional categories, representing their predictive role in the mineral formation. The non-redundant GO term sets were visualized using Revigo^35^. Intrinsic disorder regions (IDR) were predicted by an in silico analysis using flDPnn^36^, where a score above 0.3 is predicted for the region to be disordered.

### In vitro calcification experiment in the presence of SOM

A 30 cm diameter desiccator was utilized for CaCO_3_ synthesis. It contained one glass beaker (50 ml) with crushed (NH_4_)_2_CO_3_ powder covered with parafilm, punched with three-needle holes and a Petri dish containing 5 g of anhydrous CaCl_2_. They were put at the bottom of the desiccator in advance. Cellular culture microplates containing a round glass coverslip in each well were used. In each well, 750 μl of 10 mM CaCl_2_ solution was poured. In the same solution, different amounts of SOM were added to investigate its effect on CaCO_3_ formation. When CaCO_3_ overgrowth experiments were performed, the bare round glass coverslip was replaced with a round glass coverslip with its surface covered mainly by rhombohedral calcite crystals (Fig. 5). A complete description of the experimental procedures is reported in the SI.

After a four-day crystallization time, the glass coverslips were lightly rinsed with Milli-Q water, dried, and examined using an optical microscope, FTIR, Raman spectroscopy and X-ray powder diffraction (see SI). Next, the formed crystals were coated in gold and examined with a scanning electron microscope (see SI).

A preliminary set of calcium carbonate precipitation experiments were performed to define the optimal SOM concentration for the overgrowth experiments. Different SOM concentrations of 66.7 µg/ml, 33.3 µg/ml and 13.3 µg/ml were tested (see SI). The optimal one was selected based on the trade-off of having the most evident effect on calcite morphology modification compared to the control (absence of SOM) and the inhibition of the crystal growth/aggregation of the SOM (Fig. SI1–4). A concentration of 13.3 µg/ml of *O. patagonica* and 33.3 µg/ml of *S. pistillata* were selected using the data from these experiments.

## Results

### Identification of coral skeletal OM proteins

**Figure 1:**
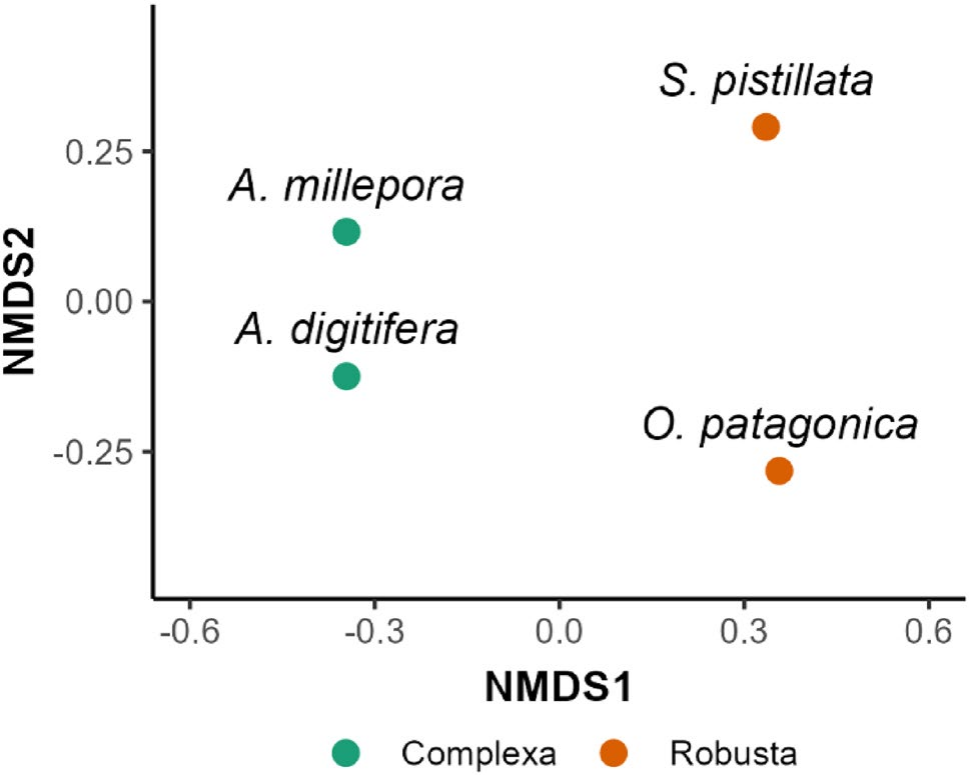
Dissimilarity in composition (Jaccard computed on generic presence-absence) between scleractinian species based on skeletal OM orthologous sequences.

We identified 73 skeletal OM proteins in *O. patagonica* using a de novo transcriptome database^27^. These proteins were compared to the published *S. pistillata, A. digitifera and A. millepora* skeletal OM proteins^14–17^. The NMDS analysis indicates a strong separation between the Robusta and Complexa species along the first axis (Fig. 1). Based on this result, we further focus our analysis on *S. pistillata*, the most similar of these species to *O. patagonica*.

**Figure 2:**
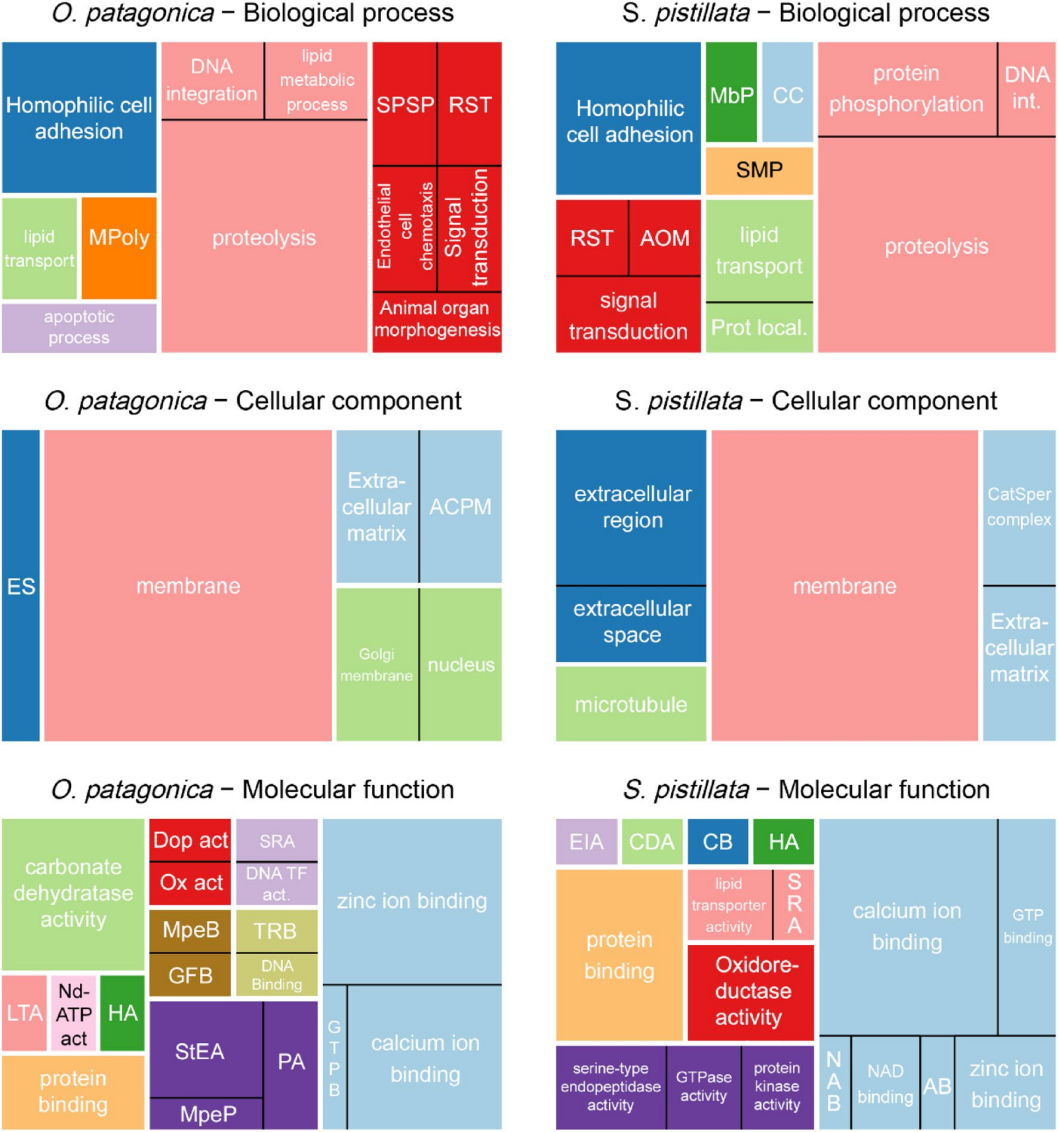
Comparison of GO term lists visualized as a treemap. Each rectangle is a single cluster representative. The representatives are joined into "superclusters" of loosely related terms, visualized with different colors. The rectangles' sizes were adjusted to reflect the frequency of representation for each species of the GO term. Abbreviations and cluster information can be found in Table SI6.

Further gene ontology (GO) analysis of the sequence composition between *O. patagonica* (*Op*-OMP) and *S. pistillata* (*Sp*-OMP) assigned 38 and 43 sequences to GO terms, respectively (Table SI4). The GO terms of the skeletal OM proteins data set are represented mainly by metabolic processes (including proteolysis and DNA integration), adhesion, cell communication, binding (including metal ion binding and protein binding) and catalytic activity (typically hydrolysis) (Fig. 2). Furthermore, the ontologies suggest key representations of proteins in the cell membrane and the extracellular space (Fig. 2). While the overall representation of the skeletal OM proteins' GO terms appear to be similar between species, the specific proteins that comprise the individual OMs are not the same. Furthermore, orthology analysis identified that 9 *Op*-OMPs share an orthologous relationship to *Sp-*OMPs, covering seven orthogroups (Tables SI4 and SI5). The shared orthogroups contain sets of MAM domain-containing proteins, cadherin proteins, acidic skeletal organic matrix proteins, ferroxidase proteins, carbonic anhydrase proteins, stereocilin proteins, and hemicentin proteins that, in turn, can be classified into three groups: (1) adhesion, (2) enzymic and (3) acidic proteins.

**Figure 3:**
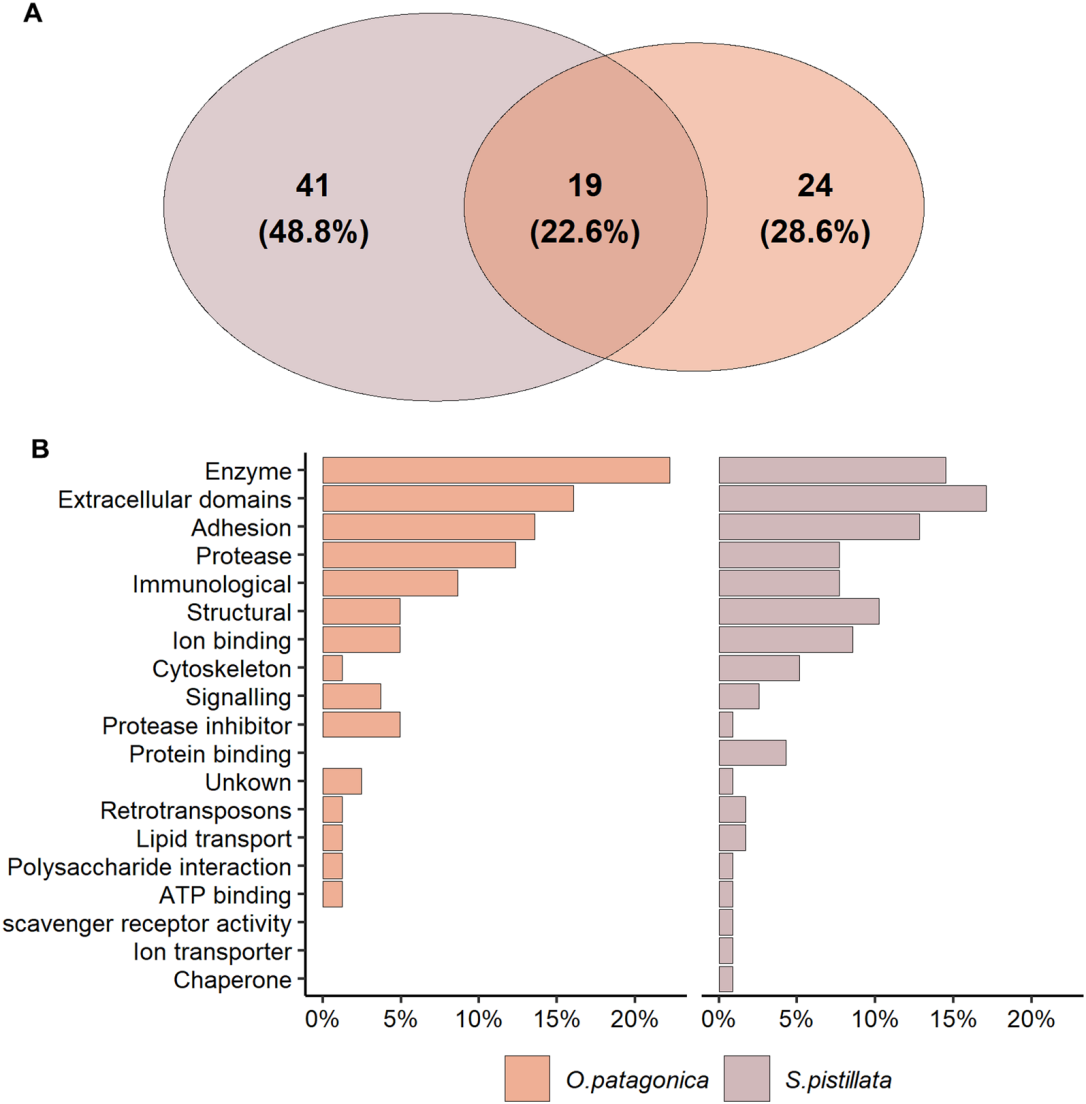
(**A**) Comparison of functional domain numbers according to the PFAM analysis. (**B**) Distribution of PFAM domain role. The values represent the percentage of each domain sharing a functional role compared to the total number of functional roles for each species.

We aimed to identify functional domains in the skeletal OM protein sequences and found 19 shared domains between both species (Fig. 3A and Table SI4). The shared domains were classified to participate in adhesion, ion binding and lipid transport. Furthermore, some appear to have an enzymic role, including proteolysis and hydrolysis, and some were annotated to be involved in the immunological response and have extracellular domains. Furthermore, while most functional domains found were species-specific (41 and 24 for *S. pistillata* and *O. patagonica*, respectively) (Fig. 3A), the domains' overall predicted roles were found to be similarly represented between the species (Fig. 3B). The most common roles of the functional domains include enzymic, proteolysis, extracellular domains, adhesive, ion binding and Immunological representing 75% and 68% of the total identified domains for *O. patagonica* and *S. pistillata*, respectively. However, certain predicted roles were identified as *S. pistillata* specific, including protein binding, scavenger receptor activity, ion transporter and chaperones.

**Figure 4:**
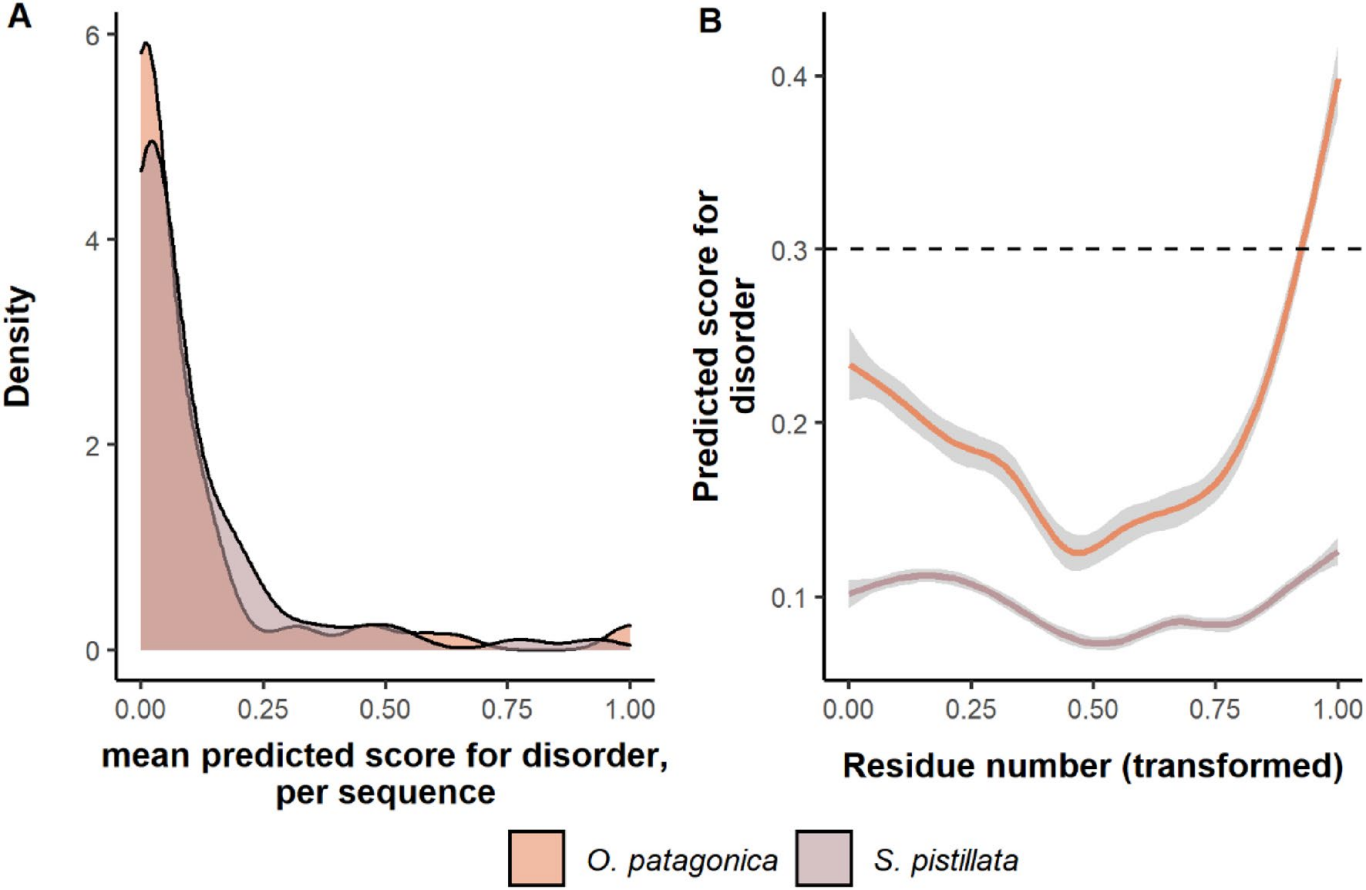
Prediction of intrinsically disordered regions. (**A**) A density plot displaying the sequences’ disorder mean predicted score per species. (**B**) The average (± standard error in grey) of the disordered score per residue across SOM proteins with at least a single region predicted to be disordered. The residue number is represented as the percentage of the entire sequence. Regions with a score above the dotted line are predicted to be disordered.

The IDR analysis revealed that both species have a mean disorder score per residue of 0.1, which signifies that they are not predicted to be intrinsically disordered. Furthermore, only two *O. patagonica* and a single *S. pistillata* skeletal OM proteins were completely intrinsically disordered. While the overall predictions appear similar between species, a Wilcoxon signed-rank test showed significantly higher IDR residues in *S. pistillata* (*p* value = 0.02). Furthermore, 80% of *S. pistillata* skeletal OM proteins are predicted to have at least a single IDR, while *O. patagonica*'s prediction is 50%. Lastly, we identify that the IDRs are predominantly found at the sequences' start and end (up to ~ 25% and from ~ 75%, respectively, of its length) for both species (Fig. 4).

### Skeletal OM proteins composition

The proteins present in the skeletal OM from *O. patagonica* have a content of Asp and Glu (52.1 mol%) higher than that present in the SOM from *S. pistillata* (30.1 mol%). The latter, however, has a higher Ser content (14.4 mol% compared to 4.5 mol% in *O. patagonica*) (Table SI1).

### In vitro overgrowth experiment

Next, we wished to test the effect of the different soluble fractions of the skeletal OMs on mineral formation. Therefore, a series of overgrowth experiments on calcite seeds were performed using the vapor diffusion method^37^ (results summarized in Table SI2).

**Figure 5:**
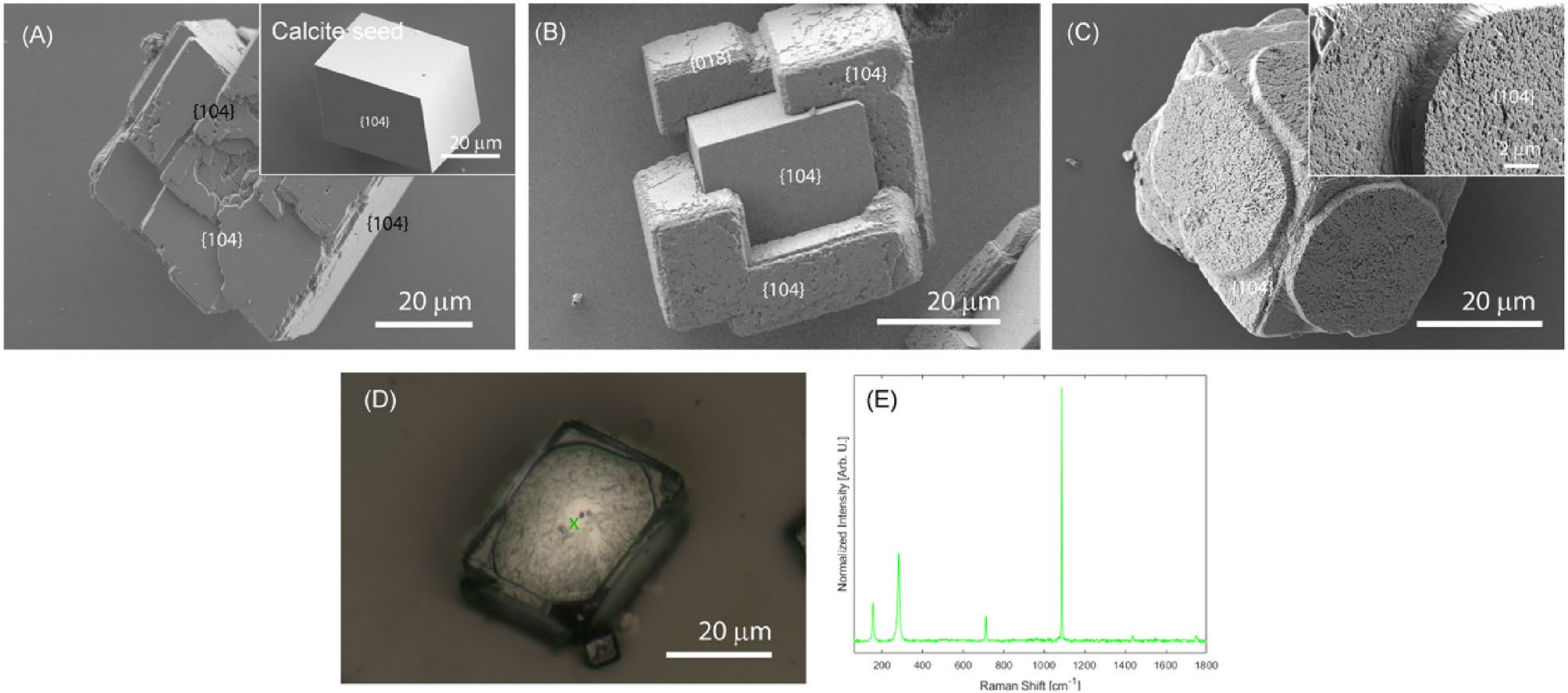
SEM images of CaCO_3_ products obtained from overgrowth experiments. (**A**) Control experiment, i.e., overgrowth experiment in the absence of soluble fraction of the skeletal OM. (**B**) Overgrowth experiment in the presence of 13.3 µg/mL of SOM extracted from *O. patagonica*. (**C**) overgrowth experiment in the presence of 33.3 µg/mL SOM extracted from *S. pistillata*. The insets show details of the overgrowth of CaCO_3_. The Miller index of the crystalline faces is reported. (**D**) Optical microscope image of an overgrown disc-like mineral on the surface of a calcite seed. (**E**) Raman spectrum collected at the point indicated by the green cross in (**D**), the vibrational absorption bands observed indicate only the presence of calcite.

The calcite seed crystals showed the typical {104} rhombohedral faces (Fig. 5A, inset). Overgrowth control experiments were also performed without SOM as control experiments. The overgrowth from the 10 mM CaCl_2_ solution induced secondary nucleation events and the deposition of additional calcite {104} layers on the calcite seeds (Fig. 5A). The effect of SOM on the CaCO_3_ overgrowth process on calcite crystal seeds was evaluated after a preliminary screening in the absence of seeds (Figs. SI1–4). The SOM extracted from *O. patagonica* and *S. pistillata* were used in the overgrowth experiments with concentrations equal to 13.3 µg/mL and 33.3 µg/mL, respectively. The products of these experiments were characterized, and each species included distinctive characteristics and mineral patterns of the overgrowth crystals. In the presence of SOM from *O. patagonica*, regular calcite crystals overgrew and covered some of the crystal edges (Fig. 5B). Their surface was irregular compared with the calcite seed's surface, showing many irregular pits. In addition, it showed {018} faces on their edges^36^. In the presence of *S. pistillata* SOM, the outcome was different. The formation of disk-like shapes centered on each {104} face of the calcite seed was observed. They uncovered the crystal edges (Fig. 5C inset) and showed the surface to be rich in irregular pits. The Raman microscopy analysis revealed that although differently shaped, this structure was calcite (Fig. 5D,E).

## Discussion

In the Scleractinia order, many species have evolved which differ in population structure, trophic strategy, macro-scale morphology, and micro-scale texture^38,39^. For each species, the skeleton's structural characteristics are a direct result of genetic control on the production of skeletal OM macromolecules^13^. These then control the calcification process, which is affected by the environment in which the coral has adapted to live^40^. To date, mineralization-associated proteins found in the skeletal OM of scleractinians were identified only in three tropical species, *S. pistillata*, *Acropora digitifera* and *A. millepora*. While those species diverged over 400 million years ago^1,6^ and belong to the Robusta and Complexa scleractinian clades, they share a similar branching growth pattern. Here we study the skeletal OM protein sequence compositions of the encrusting temperate coral *O. patagonica* belonging to the Robusta clade and compare it to previously published skeletal OM proteomes^14–17^.

Previous skeletal OM proteome analysis identified distinct sets of proteins, with only six ortholog groups across all species, termed the “core biomineralization toolkit”^19^. These proteins are predicted to have a role in the mineral nucleation, cell adhesion and structure of the organic matrix^13^. Indeed, the same core proteins are still present when cross-comparing the three tropical species with a temperate one that further differentiates in its growth pattern (Table SI5). Furthermore, our results suggest that the biomineralization mechanism is partly a function of phylogenetic proximity among species (Fig. 1). However, it is important to point out that this analysis is on a small dataset that only includes four species with high variability in their evolutionary distance (family compared to coral clades). As such, the characterization of the skeletal OM proteins of other scleractinian species from a wide array of lineages would allow for a better understanding of the disparity of proteins involved in the broader scleractinian skeleton formation.

Despite the importance of the proposed “core biomineralization toolkit”, most skeletal OM proteomes differ between the species. It was proposed that integral key molecular pathways used by skeleton-forming organisms evolved through the co-option of proteins that previously served other biological functions^41^. Interestingly, as mineralization-associated protein datasets have increased, it was found that this co-option evolution was not achieved by a common ancestor of the skeleton-forming organisms but independently within and across lineages. These findings further suggest evolutionary plasticity in which their underlying functions can be performed using a different set of proteins by different organisms, regardless of their evolutionary distance^42^.

As our results support a separation between the two coral clades (Fig. 1), we further focused our comparison on O. *patagonica’s* closest species in this dataset, *S. pistillata*. While both species belong to the Robusta coral clade, they grow under a wide range of environmental conditions^43–45^ and exhibit different growth pattern^3^.

Not all proteins present in the skeletal OM are involved in CaCO_3_ nucleation and growth processes. While random incorporation within the mineral fraction cannot be ignored, it was reported that part of the skeletal OM proteins might also regulate cell communication and the formation of the extracellular matrix^46,47^. Previous studies roughly divided the mineralization-associated proteins in metazoans into different categories. These categories include broad functional ones such as matrix formers, nucleation assisters, communicators, remodelers^46^, and functional domains such as low-complexity regions, extracellular domains, adhesion, immunological, polysaccharide interactions, enzymes, protease inhibitors and others^47^. By analyzing the specific functional annotation of both species, we identify a low rate of overlap (Fig. 3A). Nonetheless, the domains' main functionalities were similar and related to extracellular domains, immunological, enzymes and protease inhibitors (Fig. 3B). Furthermore, we found that the shared broad functional categories identified in both species are similar and relate to communication (adhesion proteins), nucleation assisters (acidic proteins) and remodelers (proteases) (Fig. 2). Regarding extracellular domains and adhesion, the most prevalent class of proteins is von Willebrand factor (vWF) proteins, which are suggested to take part in the initial mineralization process^48,49^. vWF proteins also appear to have a role in the structural organization of the organic matrix and the mineral, similar to collagens^50^. Enzymes, especially proteases and protease inhibitors, have a vital role in the remodeling of the matrix environment. One well-studied set of proteins is the carbonic anhydrases which allow for the rapid conversion of carbon dioxide to bicarbonate^51^. A sequence feature highly regarded in the protein–protein matrix and aragonitic assembly is regions of intrinsic disorder^52^ as the free energy needed to bind IDR proteins to precursor mineral is low^53^. In this study, we found a variation between species in the extent of IDR proteins (Fig. 4A) that might be associated with the difference in the quality of the skeletal OM proteome referenced sequences^54^. Yet, most skeletal OM proteins in both species were identified to have at least a single IDR, with an overall trend of these regions being found at the start and the end of the sequences (Fig. 4B). This further emphasizes that while the specific building blocks to create a skeleton are different, the overall elements that provide functionality are conserved.

The soluble fraction of skeletal OMs effect on CaCO_3_ formation was performed by in vitro homogeneous and overgrowth experiments on calcite seeds, as coral calcification starts in proximity to the larvae settlement on the substrate^55,56^. Furthermore, the best-known biochemical signals arise from mineralized crustose coralline algae that deposit calcitic structures^57,58^. The in vitro homogeneous precipitation of CaCO_3_ showed that a higher concentration of skeletal OM from *S. pistillata* is required to have a nonspecific morphological modification of calcite particles that resembles what is observed for the *O. patagonica* SOM (see SI). This different interaction capability of the SOMs with growing CaCO_3_ crystals has already been observed for other coral species having different characteristics^12,59^. A possible explanation can come from the different amino acid compositions of the two skeletal OMs. One key difference between species regards the aspartic and glutamic concentration, which was 1.7 times higher in *O. patagonica* than in *S. pistillata* SOM (SI). Usually, molecules with a high content of charged functional groups and missing a conformation interact with the growing CaCO_3_ nuclei modifying their morphology in a nonspecific way^25,60^. According to this consideration, we can suppose that the SOM macromolecules from *O. patagonica* and *S. pistillata* in a homogeneous solution do not assume a conformation. Indeed, several studies have reported that the skeletal OM molecules are intrinsically disordered in solution^53^. These observations may support the different nanoscale filling mechanisms suggested for spherulitic growth^61^. Furthermore, the authors reported a higher abundance of "sprinklers" like particles at the *O. patagonica* skeleton compared to *S. pistillata*^62^, suggesting that those "sprinklers" are the first nucleation seeds of each crystalline fiber.

The overgrowth experiments on calcite seeds confirmed the differences in mineralizers between the two Robusta SOMs. The presence of calcite seeds modifies the interaction between the SOM and the growing CaCO_3_ crystals concerning the precipitation in a homogeneous solution^63^. The results show that in the presence of the *O. patagonica* SOM, calcite crystallization occurs mainly on the edges of the seeds, which surfaces with the highest energy are found^64^. In contrast, the overgrowth of calcite in the presence of SOM from *S. pistillata* occurs at the center of {104} faces of the calcite seeds generating disk-like structures. The presence of seeds reduces the supersaturation from nucleation^65^, and the effects of the SOMs' macromolecules, or other additives, can be more evident over the growth process, which usually produces a change in crystal morphology^25,60,66^. In this context, the effect of the SOM molecules from *S. pistillata* as crystal morphology modifiers seems more specific, favoring the formation of overgrown calcite crystals in which a completely different organization (disk-like) is observed compared to those detected in the presence of the SOM from *O. patagonica* or in the control experiment in the absence of SOM molecules. However, to extensively understand the interaction between skeletal OM molecules and CaCO_3_ crystals, a study on the single molecules of the skeletal OM and their combination is necessary. This requires the not-so-easy task of their purification or biochemical expression. The information available on CARPs^50,67^ indicates that they do not assume a conformation and probably are intrinsically disordered similar to other families of highly acidic proteins^68^.

In conclusion, the observations discussed above can be contextualized in the different micro-texture of the skeleton of the two coral species. Furthermore, the knowledge gained both from the proteomic analysis and these in vitro experiments on the calcification process in coral can be integrated with the already available information on the molecular toolkit that controls the calcification process^13,50^ and on the role of single proteins^69–72^ in addressing the pathway of the mineralization process. Although the proposed "core biomineralization toolkit"^19^ and the skeletal OM proteome set of functional categories are conserved across scleractinians, this study emphasizes that each species utilizes its own specific elements to achieve the conserved functional category. This suggests that the early diversification^1,6^ allowed species-specific adaptations to diverse environmental conditions. Therefore, with an eye on better understanding the ability of stony corals to calcify under diverse environmental conditions, we need to better explore the differences in those categories and across a wide range of species.

## Supplementary Information

Supplementary Information 1.

Supplementary Information 2.

Supplementary Information 3.

Supplementary Information 4.

Supplementary Information 5.

## Data Availability

All alignments, trees, and protein sequences used for orthology inference are available on GitHub (https://github.com/Mass-Lab/Zaquin_Op_Sp_SOM_comparison.git) and are publicly available. The datasets generated during the current study are publicly available in the ProteomeXchange repository under file number PXD034601 (http://proteomecentral.proteomexchange.org/cgi/GetDataset?ID=PXD034601).
