# Supplementary material for: Different skeletal protein toolkits achieve similar structure and performance in the tropical coral Stylophora pistillata and the temperate Oculina patagonica
Source: Sci Rep. 2022 Oct 4;12:16575. doi: 10.1038/s41598-022-20744-0 (PMC9532382; doi:10.1038/s41598-022-20744-0)
Supplement: Supplementary file 1 — Supplementary Information 1. [file 41598_2022_20744_MOESM1_ESM.docx]

Supporting Information

# Extraction of the Organic Matrix

The soluble organic matrix was extracted through decalcification using a 0.1 M CH_3_COOH solution, as previously reported (Reggi et al. 2008). The SOM concentration was expressed as amount of protein from the amino acid analysis.

# Protein Sequencing

O. patagonica skeletal protein samples were sequenced at the De Botton Protein Profiling Institute at the Weizmann Institute of Science, Rehovot, Israel. Skeletal protein samples were dissolved in 5% SDS and digested with trypsin using the S-trap method overnight at room temperature. The resulting peptides were analyzed using a nanoflow ultra-performance liquid chromatography (nanoAcquity) coupled to a high resolution, high mass accuracy mass spectrometer (Fusion Lumos). The sample was trapped on a Symmetry C18 0.18*20 mm trap column (Waters, Inc) and separated on an HSS T3 0.075*250 mm column (Waters, Inc.) using a gradient of 4–28% (80% acetonitrile, 0.1% Formic acid) for 150 min. The spray voltage was set to +2kV. The data were acquired in the Fusion Lumos using a Top Speed Data-Dependent Acquisition method using a cycle time of 3s. A mass spectrometry (MS) 1 scan was performed in the Orbitrap at 120,000 resolutions with a maximum injection time of 60ms. The data were scanned between 300 and 1800 m/z. MS2 was selected using a monoisotopic precursor selection set to peptides, peptide charge states set to +2 to +8 and dynamic exclusion set to 30s. MS2 was performed using Higher-energy C-trap dissociation (HCD) fragmentation scanned in the Orbitrap, with the first mass set to 130 m/z at a resolution of 15,000. Maximum injection time was set to 60ms with automatic gain control of 5×10−4 ions as a fill target.

# SOM Amino acid composition analysis

Amino acid analysis was conducted by Ultra High-Performance Liquid Chromatography (UHPLC Agilent Technologies) equipped with a diode array detector (Agilent Technologies). Macromolecules were hydrolyzed using 6 m HCl for 24 h at 100°C. During hydrolysis, complete or partial destruction of several amino acids occurs: tryptophan is destroyed, and serine and threonine are partially destroyed. Sulphur amino acids are altered. Then samples were dried and used for derivatization with 6-Aminoquinolyl-N-hydroxysuccinimidyl carbamate (AQC). Detection occurred at a wavelength of 260 nm.

# Calcium carbonate seed crystal syntheses

A 30cm diameter desiccator was utilized for seeds synthesis. It contained one glass beaker (50mL) with crushed (NH_4_)_2_CO_3_ powder covered with parafilm, punched with three needle holes and a Petri dish containing 5g of anhydrous CaCl_2_. They were put at the bottom of the desiccator in advance. Microplates for cellular culture containing a round glass coverslip in each well were used. In each well, 750 μL of 10 mM CaCl_2_ solution were poured. After a crystallization time of four days the glass coverslips were lightly rinsed with milli-Q water, dried, and examined using an optical microscope. The crystals on some coverslip, after gold coating, were observed with a scanning electron microscope.

# Raman spectroscopy

Raman measurements were conducted on a LabRAM HR Evolution instrument (Horiba, France). The instrument is equipped with an 800 mm spectrograph which allows for sub-two wavenumber pixel spacing when working with 600 grooves/mm grating at 532 nm excitation. The samples were exposed to the laser light by a ×50 LWD NA=0.5 objective (LMPlanFL N, Olympus, Japan). The LabRAM instrument has a 1024 × 256 pixel, an open-electrode, front-illuminated, cooled CCD camera. The system is set around an open confocal microscope (BX- FM Olympus, Japan) with a spatial resolution better than 2µm using the ×50 objective. Exposure was set according to the signal intensity, and exposures between 15 seconds and 1 minute were used. This system is equipped with ultra-low frequency capability, four laser lines many objectives, and several gratings to allow modular and flexible use for samples of significant variability.

# Characterization of CaCO_3_ precipitates

The optical microscope observations of CaCO_3_ precipitates were made with a Leica microscope equipped with a digital camera. The scanning electron microscopy image were acquired using a Leica Cambridge Stereoscan 360 scanning electron microscope. The samples were gold coated (2 nm) before their observation. A Thermo Scientific™ Nicolet™ iS™10 FTIR Spectrometer was used to collect the FTIR spectra. Disk sample for FTIR analysis was obtained by mixing a small amount (< 1 mg) of product with 100 mg of KBr and applying a pressure of 45 tsi (620.5 MPa) to the mixture using a press. X-ray diffraction patterns were collected using a PanAnalytical X’Pert Pro diffractometer equipped with a multi-array X’Celerator detector using Cu Kα radiation generated at 40 kV and 40 mA (λ = 1.54056 Å). The diffraction patterns were collected in the 2θ range between 20° and 60° with a step size (Δ2θ) of 0.02° and a counting time of 100 s.

**Table SI1:** Amino acid composition (mol%), expressed as a percentage of the amino acids present in the SOM extracted from *Stylophora pistillata* and from *Oculina patagonica*. On the right a histogram showing the molar percentage of amino acids present in the SOM of *Stylophora pistillata* (blue) and *Oculina patagonica* (green).


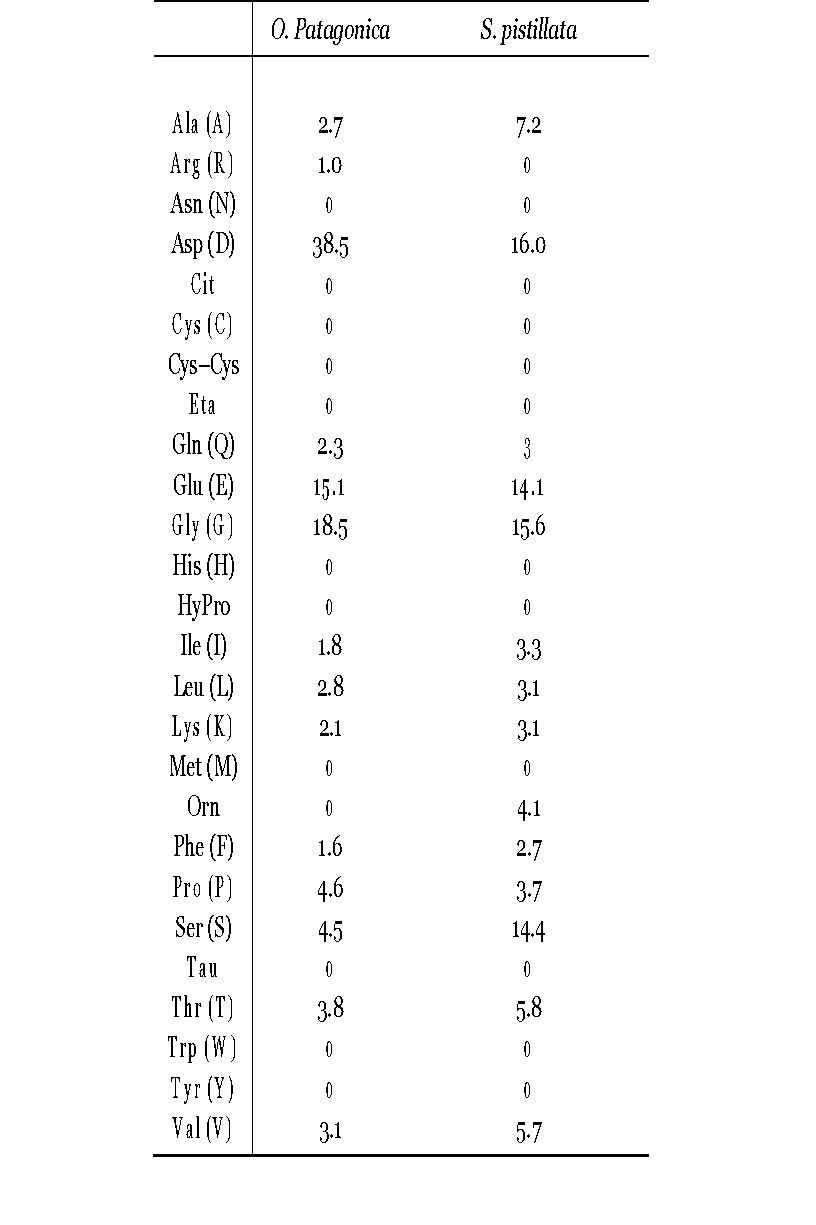

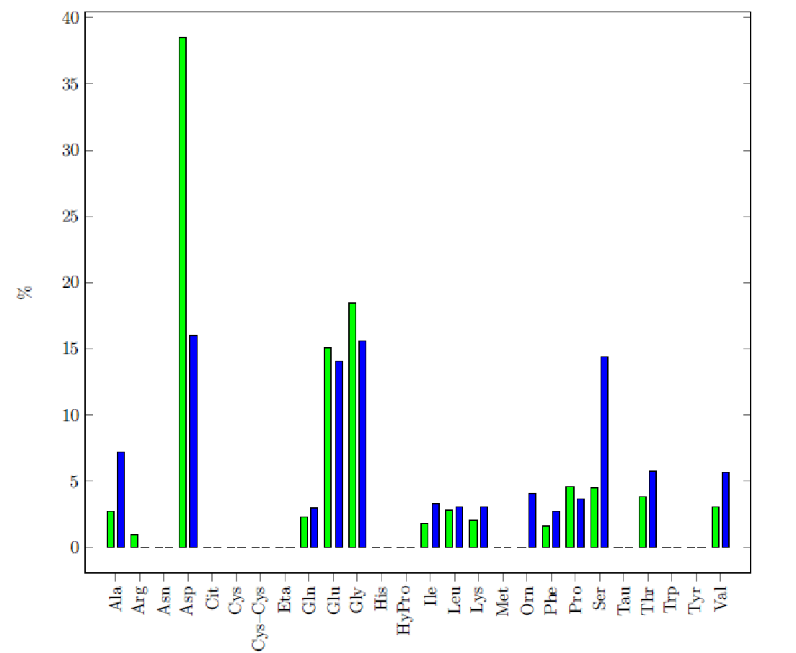


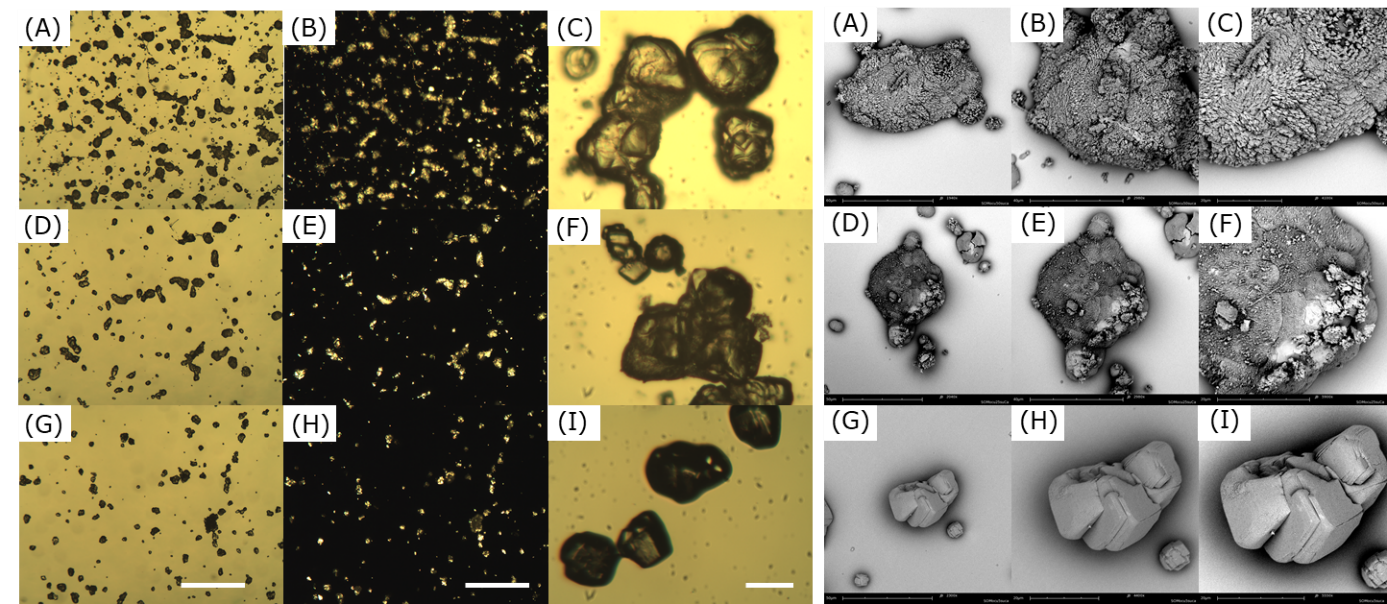
**Figure SI1**. Homogeneous calcium carbonate crystallization in the presence of SOM extracted from the skeleton of *O. patagonica* or *S. pistillata*. (*left*) Optical microscope images of the precipitates obtained from a 10 mM CaCl_2_ solution in the presence of SOM from *O. patagonica* (A – C) 66.7 µg / mL; (D – F) 33.3 µg / mL; (G – I) 13.3 µg / mL. The images (A), (B), (D), (H), (G), (E) are with a magnification of 4x, while (C), (F), (I) with a magnification of 32x. Images (B), (H), (F) were obtained with polarized light. (*right*) SEM images of precipitates obtained from a 10 mM CaCl_2_ solution in the presence of SOM from *O. patagonica*: (A – C) 66.7 µg / mL; (D – F) 33.3 µg / mL; (G – I) 13.3 µg / mL.

**Figure SI2**. Normalized FTIR spectra (left) and X-ray powder diffraction patterns of precipitated obtained from a 10 mM CaCl_2_ solution in the presence of SOM from *O. patagonica* (a) 66.7 µg / mL; (b) 33.3 µg / mL; (c) 13.3 µg / mL. The diffraction peaks with the relative Miller indices are indicated according to the reference pattern of the calcite PDF 00–005–0586.
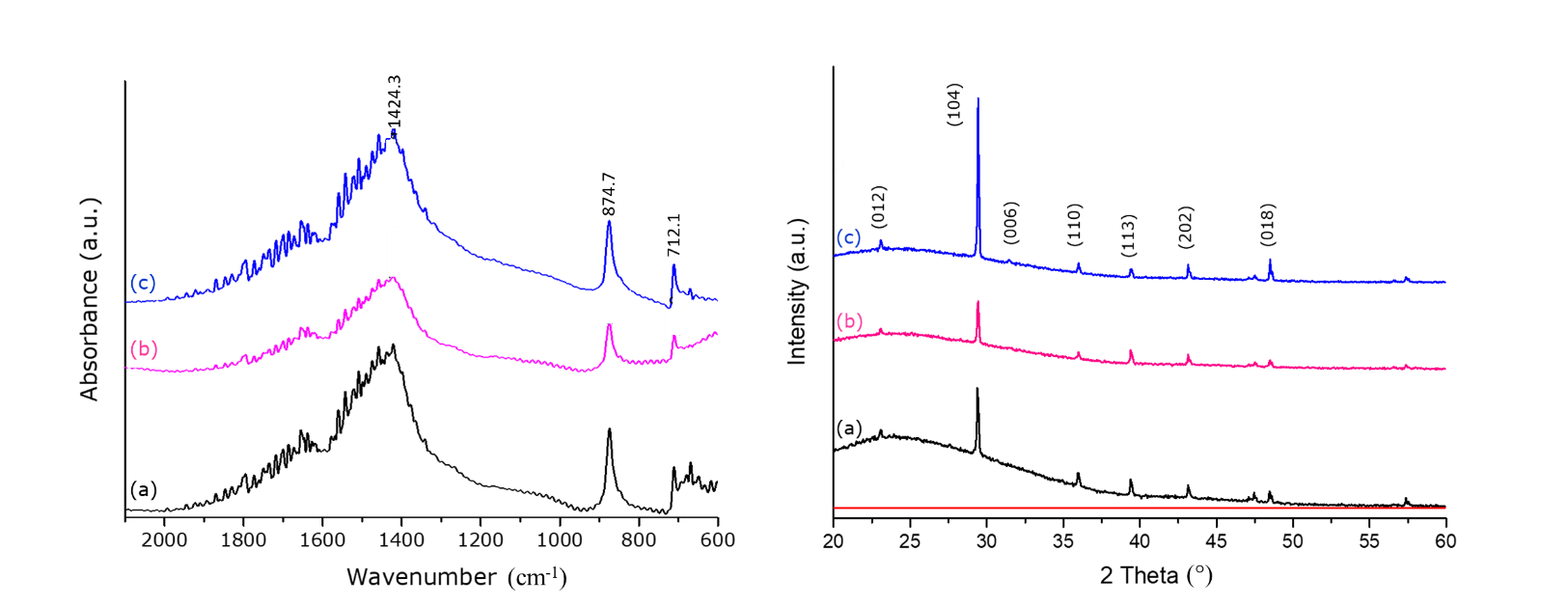


**Figure SI3**. (*left*) Optical microscope images of the precipitates obtained from a 10 mM CaCl_2_ solution in the presence of SOM from *S. pistillata* (A – C) 66.7 µg / mL; (D – F) 33.3 µg / mL; (G – I) 13.3 µg / mL. The images (A), (B), (D), (H), (G), (E) are with a magnification of 4x, while (C), (F), (I) with a magnification of 32x. Images (B), (H), (F) were obtained with polarized light. (*right*) SEM images of precipitates obtained from a 10 mM CaCl_2_ solution in the presence of SOM from *S. pistillata*: (A – C) 66.7 µg / mL; (D – F) 33.3 µg / mL; (G – I) 13.3 µg / mL.
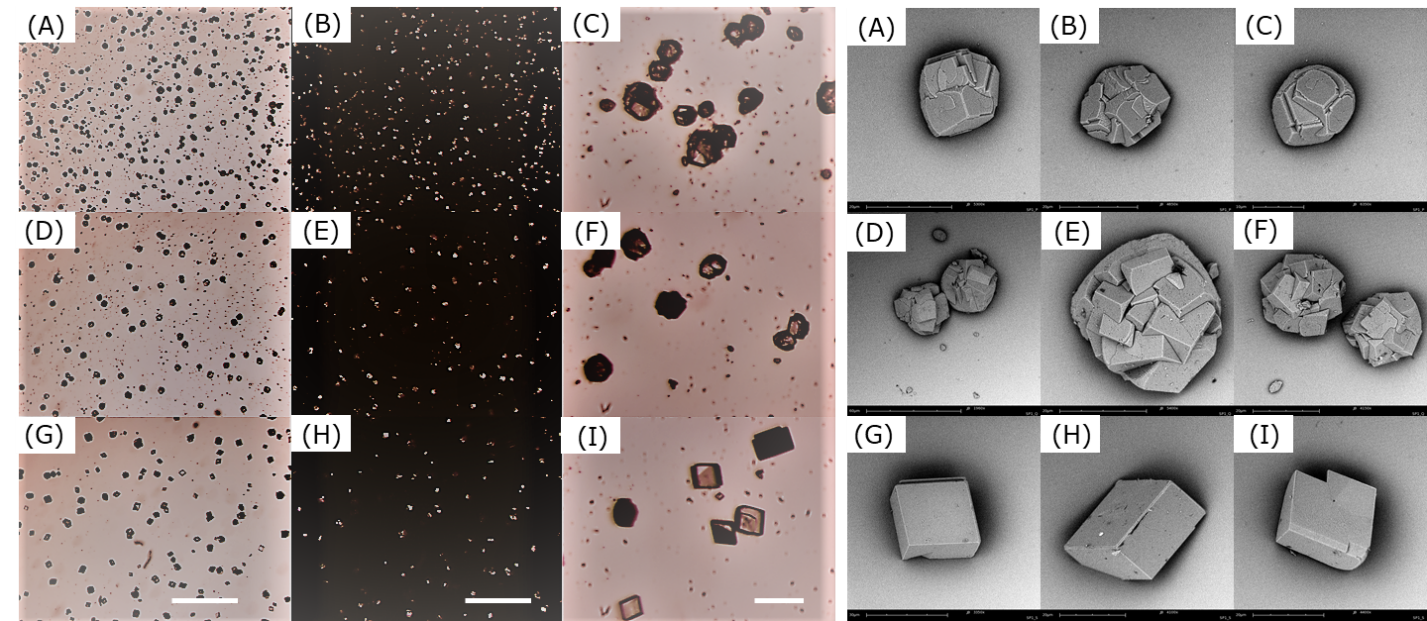


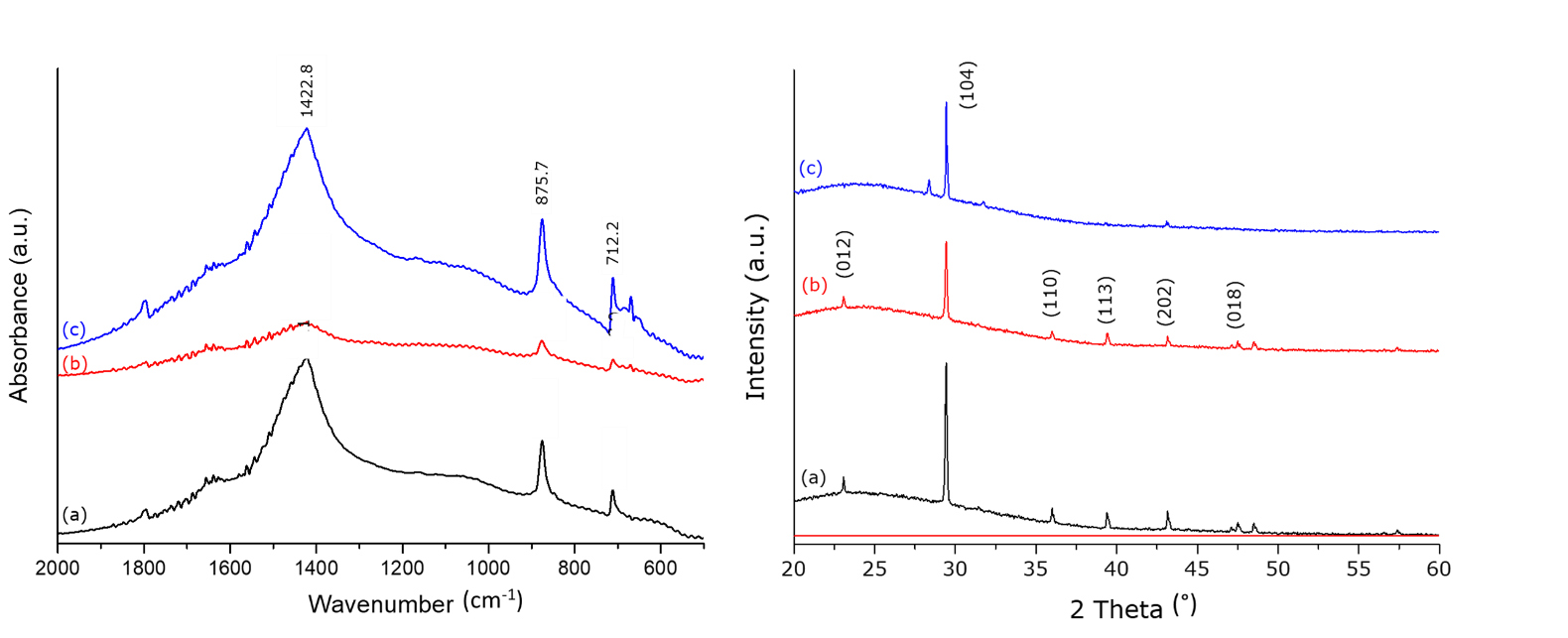
**Figure SI4**. Normalized FTIR spectra (left) and X-ray powder diffraction patterns of precipitated obtained from a 10 mM CaCl_2_ solution in the presence of SOM from *S. pistillata* (a) 66.7 µg / mL; (b) 33.3 µg / mL; (c) 13.3 µg / mL. The diffraction peaks with the relative Miller indices are indicated according to the reference pattern of the calcite PDF 00–005–0586.

**Table SI2**: Summary of the results obtained from the overgrowth experiments on calcite seeds from a 10 mM CaCl_2_ solution in the presence of seeds and SOM extracted from *O. patagonica* or *S. pistillata*.

| Coral species | Overgrowth phase | Shape of the overgrowth phase |
| --- | --- | --- |
| Control* | C | {104}; rh. |
|  |  |  |
| O. patagonica | C | {018}, {104}; rh. ass. rough |
|  |  |  |
| S. pistillata | C | disk. rough |

* No SOM was present in the precipitating solution. C indicates calcite. The shape of the crystals has been defined as follows: rh = rhombohedral; disk = disk-like structure.

**Table SI3**. Species list used for the phylogenetic reconstruction analysis.

**Table SI4.** OM proteins from *O. patagonica* and *S. pistillata*. The table list their orthology inference; assigned orthogroup and orthologous relationship between sequences. The table further list each sequence functional classification derived from our analyses.

**Table SI5.** Orthologous relationship of the stony corals' sequences. Each row in a file contains the gene(s) in one species that are orthologues of the gene(s) in the other species.

**Table SI6.** Clusters information and abbreviation used in displaying the Revigo figure (Figure 1).
